# Modernising Hip Fracture Surgery

**DOI:** 10.2174/1874325001711011179

**Published:** 2017-10-31

**Authors:** Dr. Hannah Dawe

**Affiliations:** St Georges Hospital, Tooting, SW170QT, UK

Edward Dawe FRCS(Tr & Orth) BSC(Hons) Dip(SEM), Specialty Registrar Year 8, Royal Surrey County Hospital Guildford, Honorary Clinical Lecturer Brighton and Sussex Medical School, ORKSS research collaborative Piers Page MPhil MRCSEd, Specialty Registrar Year 4, Frimley Park Hospital, Honorary Clinical Lecturer Brighton and Sussex Medical School, ORKSS research collaborative Hip fracture, while affecting a broad group of patients including the relatively young and extremely active, is a disease of old age and for some patients it represents an end of life event. The management of these injuries is challenging, as the highest standard of evidence is required and best practice should be agreed, but nonetheless each case must be judged on its individual merits. Out of this is being born a truly multidisciplinary science of hip fracture care which is both multi-faceted and increasingly detailed.

Historically, hip fracture surgery was delegated to the most junior surgeons with little or no emphasis on perioperative analgesia, rehabilitation, management of comorbidities or secondary prevention. As we now understand the mortality and quality of life impact on patients, the economic burden on society and the complexity of caring for these patients, this practice is rightly shifting towards a consultant-led service based on timely, evidence-based interventions.

While recent years have seen an increase in total hip arthroplasty for intracapsular fracture, the mainstay of treatment remains the hemiarthroplasty. In this issue Jones reviews factors predicting one of rare but problematic complications, dislocation.

Dawe *et al* present and summarize the evidence from large studies of specialist anaesthesia for hip fracture, considering important questions such as the role of intra-operative hypotension in perioperative mortality, the association between spinal anaesthetic dose and mortality and thresholds for transfusion. Also covered is the important topic of communication impaired by dementia and its impact on accessibility of pain relief to these patients.

The relatively new sub-speciality of orthogeriatrics has effected a landmark change in hip fracture care; elderly care physicians supervise the pre-operative management of these patients, help liaise with relatives, guide rehabilitation after surgery and help provide expert input to end of life decision-making. Helen Wilson's article discusses the advent of this specialty and how this model of care provides tangible benefit to patients.

Jacob *et al* discuss decision-making in extracapsular fractures and present the evidence behind dynamic hip screw (DHS) and intramedullary nail constructs. In a second article, the same authors provide valuable technical tips for surgeons of all level who use these devices in their practice. Stott’s ‘What I do’ piece provides an invaluable and insightful guide to the strategy he uses when faced with failed operations for hip fracture.

Saini *et al.* report the extremely low vitamin D levels in patients admitted to their unit with a hip fracture in their audit of calcium and vitamin D supplementation after hip fracture. Further findings relating to the effect and uptake of secondary prevention medication are thought-provoking and beg the question of whether we need to re-think strategy at a more fundamental level.

We commend these authors’ work to you and are confident it will help advance practice in this modern orthopaedic epidemic.

